# The Singleton Fallacy: Why Current Critiques of Language Models Miss the Point

**DOI:** 10.3389/frai.2021.682578

**Published:** 2021-09-07

**Authors:** Magnus Sahlgren, Fredrik Carlsson

**Affiliations:** ^1^ AI Sweden, Stockholm, Sweden; ^2^ RISE, Stockholm, Sweden

**Keywords:** language models, natural language understanding, representation learning, neural networks, meaning

## Abstract

This paper discusses the current critique against neural network-based Natural Language Understanding solutions known as *language models*. We argue that much of the current debate revolves around an argumentation error that we refer to as *the singleton fallacy*: the assumption that a concept (in this case, language, meaning, and understanding) refers to a single and uniform phenomenon, which in the current debate is assumed to be unobtainable by (current) language models. By contrast, we argue that positing some form of (mental) “unobtanium” as definiens for understanding inevitably leads to a dualistic position, and that such a position is precisely the original motivation for developing distributional methods in computational linguistics. As such, we argue that language models present a theoretically (and practically) sound approach that is our current best bet for computers to achieve language understanding. This understanding must however be understood as a computational means to an end.

## 1 Introduction

We are at an inspiring stage in research on Natural Language Understanding (NLU), with the development of models that are capable of unprecedented progress across a wide range of tasks (Wang et al., 2019). At the same time, there are critical studies being published that demonstrate limitations of our current solutions (McCoy et al., 2019; Niven and Kao, 2019; Ribeiro et al., 2020), and more recently, voices have been raised calling for, if not taking a step back, then at least to stop for a moment and recollect our theoretical bearings (Bender and Koller, 2020; Bisk et al., 2020). Even if these latter theoretical contributions have slightly different perspectives—Bisk et al. (2020) introduce the notions of *World Scopes* as a way to argue for the futility of using only text data to train NLU models, while Bender and Koller (2020) posit a strict distinction between form and meaning, arguing that models only trained on form cannot grasp meaning—they share what we consider to be a healthy skepticism of the currently somewhat opportunistic and methodologically narrow-minded development.

The main controversy in this recent debate is the question to what extent our current NLU approaches—i.e., predominantly Transformer neural network language models—can be said to really *understand* language, and whether the currently dominating research direction has any potential at all to lead to models with “real” understanding. To put the point succinctly: will it eventually prove to be enough to train a thousand-layer quadrillion-parameter Transformer language model on the entire world’s collected texts, or do we need something *more* or something *else* to reach true NLU? And what is “true” NLU anyway—perhaps there is no such thing? The recent excitement and hype in news and popular science press surrounding GPT-3 [see, e.g., Thornhill (2020) and Marr (2020)] of course does nothing to dampen this controversy. While we fully share the assessment that more theoretical considerations would be beneficial for current and future NLU development, we think that both Bisk et al. (2020) and Bender and Koller (2020) oversimplify important core discussion points. These simplifications admittedly serve a rhetorical purpose, making the arguments come across as convincing by appealing to readers’ intuition, but they obscure the inherent complexity of notions such as meaning and understanding, thereby risking to mislead, or at least oversimplify, the important questions. Our contribution therefore aims to analyze, and hopefully clarify, some of these arguments while also raising some novel discussion points of its own.

While most other commentators, such as Bisk et al. (2020) and Bender and Koller (2020), express skepticism towards the ability of language models to understand language, we aim to provide a slightly different perspective, focusing on the question what it means to *understand language*. Our position is that “understanding” does not refer to a single, well-defined phenomenon, and that the ability to identify and manipulate the symbols and structures that constitute the language system must be contained within a coherent account of language understanding.

## 2 Language, Meaning, and Understanding: A Philosophical Trifecta

It is always precarious to build arguments on inherently vague and general concepts such as “language,” “understanding,” and “meaning,” since the resulting theoretical constructs become so overly general that they almost become vacuous. We think that this is precisely what cumbers the current debate, and our aim in this paper is to shed light on some of the inherent challenges with using these concepts to problematize the current development in NLU. In this first section, we discuss how these terms are used in the current debate, and we argue that most of the current critique of the semantic capabilities of language models rest on a misunderstanding, or misrepresentation, that we refer to as *the singleton fallacy*. In short, this argumentation error consists in assuming that a term refers to a single uniform phenomenon, when in practice the term can refer to a large set of vaguely connected phenomena. Sections 2.1 and 2.2 discusses the concepts of “language” and “understanding,” while Section 2.3 focuses on how current language models understand language.

### 2.1 Language is Not One Single Thing

Language is normally defined as the system of symbols that we use to communicate, and learning a language entails learning the set of symbols and rules that define the system. Learning the set of symbols corresponds to vocabulary acquisition, while learning the rules entails recognizing and formalizing grammatical, morphological, and syntactic regularities. We measure these competencies in humans—often indirectly—by using various language proficiency tests, such as vocabulary tests, cloze tests, reading comprehension, as well as various forms of production, interaction, and mediation tests (such as translation and interpretation). To evaluate our current NLU solutions, we often use specifically designed test sets, such as GLUE (Wang et al., 2018), SuperGLUE (Wang et al., 2019) and Dynabench (Nie et al., 2020), or more specific probing tasks that attempt to more directly measure a model’s capacity to represent a specific linguistic phenomenon (Jawahar et al., 2019; Liu et al., 2019; Tenney et al., 2019). Even if current language models have been shown to underperform in some specific test settings [such as their ability to handle negation (Ettinger, 2020)], there is an overwhelming body of empirical results to demonstrate that current language models have a passable capacity to detect the symbols and rules that define human language.

But this is not what is under dispute in the current debate; what *is* under dispute is whether the continuously increasing test scores really indicate progress towards “understanding,” or if the currently dominating paradigm of (predominantly text-based) language modeling leads to a dead end, populated with gigantic models that simply trade one sequence for another (i.e., models that only know about the symbols and rules of the language system). In other words, does structural knowledge about the workings of the language system suffice, or does language understanding require additional competencies and inputs?

Of course, the question is: suffice for what? Presumably, we develop NLU systems in order to do the things we humans do with language. And here is the complication; we do not only do *one* thing with language. We humans do lots of different things with language, ranging from primal vocal expressions, over basic naming of objects and responding to simple questions, to more complicated tasks such as following instructions, arguing, or participating in negotiations. Language behavior is decidedly not one single activity, but a collection of many interrelated competencies and activities that together constitute the totality of (human) linguistic behavior. Wittgenstein (1953) refers to the relations between these interrelated linguistic activities as *family resemblances*, and he explains the situation thus: “Instead of producing something common to all that we call language, I am saying that these phenomena have no one thing in common which makes us use the same word for all—but that they are related to one another in many different ways. And it is because of this relationship, or these relationships, that we call them all language” As we know, Wittgenstein used the term “language game” to refer to linguistic activities connected by such family resemblances.

The game analogy is fitting to describe human linguistic behavior. We have all been in situations where we encounter new language games (think about trying to buy something from a specialized store without any prior knowledge of the domain), with crippling disability to participate in the language game, despite being a proficient native speaker of the language in question. All humans have a slightly different set of linguistic abilities, and even if two language users share a linguistic ability—e.g., arguing—they are typically not equally good at it. Linguistic proficiency is a continuous scale, ranging from more or less complete incompetence to more or less complete mastery. We normally do not think about (human) language learning and linguistic proficiency as a pursuit of one single ultimate goal, whose completion is a binary outcome of either success or failure, but rather as a collection of tasks that can be performed in a number of different ways. When trying to define a criteria for quantifying linguistic proficiency, we must acknowledge that a totality of these linguistic skills is never actually manifested (as far as we know) in one single human language user. All current language learning tests are modelled according to this assumption, and hence deliver scalar results on a number of different test settings.

The fact that linguistic competence is not a binary phenomenon is not necessarily a point that is in contention, but we think that recognizing the multifarious nature of human language behavior alludes to an important point: rather than asking whether a language model understands or not, we should ask *to what extent*, and *in which way*, a model understands. Framing the question in this way will also foster more realistic expectations on NLU solutions. Instead of demanding them to be flawless, generic and applicable to every possible use case, we may be better off adopting the same type of expectations as we do with human language users; they will all be different, and—importantly—good at different things (and, in the case of humans, good at different things *at different times, and in different situations*).

### 2.2 How Should We Understand “Understanding”?

The main controversy in the current debate is not so much whether language models can be trained to perform various types of language games fairly well (most commentators seem to agree that they indeed can). The main controversy is instead whether a language model that has been trained to perform some language game actually possesses any *real* understanding of language. Most commentators are critical in this respect; Bisk et al. (2020) argue that “meaning does not arise from the statistical distribution of words,” and Bender and Koller (2020) claim that “the language modeling task, because it only uses form as training data, cannot in principle lead to learning of meaning.”

For both authors, it seems that linguistic proficiency—being able to play some language game—is not enough to grant the model the ability to really understand language. So what is needed? And what do these commentators *mean* by understanding? The problematic nature of the term “understanding” is demonstrated by the utter definitional extremes that can be found in the current debate. On the one hand, we have Bisk et al. (2020), who do not provide any concrete definition of what they mean by understanding, and instead simply appeal to intuition. On the other hand, we have Bender and Koller (2020), who provide an admirably clear and concise definition of understanding as referring “to the process of retrieving intent *i* given expression *e*”.

In traditional language philosophy and linguistic theory, one normally distinguishes between three different types of understanding of a given linguistic expression. One type is the intra-linguistic, structural type of understanding that enables the subject to produce coherent linguistic output. Another is the referential understanding that enables the subject to identify (and visualize) corresponding things and situations in the world, and a third is the social understanding that enables the subject to interpret other peoples’ intentions. These different types of understanding map approximately to Bisk et al.’s World Scopes 1–3 (intra-linguistic understanding), 4 (referential understanding), and 5 (social understanding). In more traditional linguistic terms, we might use the terms *conventional* meaning, *referential* meaning, and *pragmatic* meaning to refer to these different types of information content. So while Bisk et al. (2020) cover the entire spectrum of understanding with their World Scopes, the definition of understanding proposed by Bender and Koller (2020) primarily applies to pragmatic aspects of language use, where the task of the interlocutor is to identify the intents of the speaker (or writer).

### 2.3 The Structuralism of Contemporary Natural Language Understanding Approaches

It may be useful at this point to consider how (one breed of) current NLU systems “understand” language. A particularly successful approach to NLU at the moment is to use deep Transformer networks that are trained on vast amounts of language data using a language modeling objective, and then specialized (or simply applied) to perform specific tasks (Collobert and Weston, 2008; Peters et al., 2018; Devlin et al., 2019; Brown et al., 2020). Such models implement a fundamentally structuralist—and even *distributionalist* (Sahlgren, 2008; Gastaldi, 2020)— view on language, where a model of how symbols are combined and substituted for each other is learned by observing samples of language use. The language modeling component (or, in somewhat older methods, the embeddings) encodes basic knowledge about the *structure* of the language system, which can be *employed* for solving specific linguistic tasks. This is eminently well demonstrated in the recent work on zero-shot learning (Yin et al., 2019; Brown et al., 2020).

The “understanding” these models have of language is entirely structural, and thus does not extend beyond the language system—or, more accurately, beyond the structural properties of the input modality. Speaking in terms of the different types of understanding we discussed above, this refers to conventional, intra-linguistic understanding. Note that this is an intentional restriction, since the learning objective of these models aim to capture distributional regularities. It follows that if the input signal consists of several different modalities (e.g., language *and* vision, sound, touch, and maybe even smell and taste), then the resulting structural knowledge will cover *all* of these modalities. A distributional model built from multimodal data will thus be able to employ its (structural) knowledge cross-modally, so that, e.g., vision data can affect language knowledge and vice versa (Wang et al., 2019; Su et al., 2020). Such a multimodal model may be able to form images of input text, so that when given an input such as “door” it can produce, or at least pair the text with, an image of a door (Ramesh et al., 2021). There have also been a fair amount of work on image captioning, where a model produces text based on an input image (Herdade et al., 2019; Li et al., 2019; Radford et al., 2021).

This cross-modal ability cannot be described as purely intra-linguistic, since it covers several modalities. While we might not want to go as far as to call this referential understanding, it should certainly count as *visual* understanding (of language). It is an interesting question how we will view distributional models when we start to incorporate more modalities in their training data. What type of understanding would we say a language model has if it can connect linguistic expressions to actions or situational parameters? Multimodality is mentioned by both Bisk et al. (2020) and Bender and Koller (2020) as a promising, if not necessary, research direction towards future NLU, and we agree; it seems like an unnecessary restriction to only focus on text data when there is such an abundance of other types of data available. However, there is also a more fundamental question in relation to multimodality, and that is whether there are things that *cannot* be learned (about language) by merely reading large bodies of text data?

## 3 There is Nothing Outside the Text

The last section problematized the use of philosophically nebulous terms such as “understanding” and “meaning,” and we argued that careless use of such terms invites to an argumentation error, or oversimplification, that we call the singleton fallacy. In this section, we discuss some of the more concrete arguments against language models, and we argue that they inevitably collapse into dualism, which we consider to be a defeatist position for an applied computational field of study. A consistent theme in the current critique of language models is the assumption that text data is insufficient as learning material in order to reach real understanding of language. We have already noted the perils of using such general statements as “understanding of language,” but in this section we will take a closer look at some of the specific arguments and thought experiments that motivate this assumption.

### 3.1 The Symbol Manipulators: Octopuses and Language Models

Both Bisk et al. (2020) and Bender and Koller (2020) clearly think that text data is insufficient to reach true NLU. Bisk et al. (2020) refer to the meaning of “painting” as a case in point when a purely linguistic signal will be insufficient to learn “the meaning, method and implications” of the concept. It is not clear to us that this is the case; learning from language *is* learning about our world; we use language to communicate and store experiences, opinion, facts and knowledge. Bender and Koller (2020) constructs a more elaborate thought experiment to make basically the same point. The thought experiment features a hyper-intelligent octopus (“O”) that inserts itself in the middle of a two-way human communication channel, and that eventually (due to loneliness, we are told) tries to pose as one of the human interlocutors. The conclusion of this thought experiment is that O would fail to respond adequately when faced with an unfamiliar situation (being asked to produce plans for constructing an anti-bear weapon from sticks), and that all “successful” communication prior to this only made sense to the human receiver because the human assumed meaning in the previous communication.

We are not convinced by this “octopus test.” Although we find it intuitive that O (assumed to not have had access to text for similar situations) would fail to produce an answer solving the problem at hand, we find it equally intuitive that a human who is not a professional bear fighter would produce an similarly unhelpful answer.^1^ Therefore, we disagree with the conclusion that any failure from O is a direct result of it being exposed to text only. Indeed we fail to see why O would be unable to produce a “successful” answer from which the human can derive meaning. A response such as *“I’m sorry I have no idea”* or *“What?”* admittedly does not solve the urgent bear problem, but could still be argued to pass as meaningful, and can certainly be argued to be within the capabilities of our text-constrained hyper-intelligent-deep-sea-octopus.

Putting the point concisely, the “octopus test” seemingly intertwines the lack of expertise with an innate limitation caused by the text modality constraint. Although lack of expertise and knowledge points to a lack of understanding, it is not a constraint imposed only on language models. Any entity, regardless of what modalities its reality is confined to, can theoretically find itself in situations where it lacks experience, and therefore lacks expertise. Hence, we think that a more convincing argument is required in which experience about the topic is more clearly separated from the distinction between form and meaning.

The insufficient distinction between the lack of shared experience and the incapability of shared experience due to lacking modalities, also frames our core criticism of Bisk et al. (2020). It is our understanding that Bisk et al. (2020) similarly argues that text-only models will fail, not only due to a lack of shared experience, but that operating solely with text inherently prevents these shared experiences. This intuitively makes sense, but we are yet not convinced by any provided examples where additional modalities would be required in order to participate in “successful” communication. This also applies to the “Java-code example” of Bender and Koller (2020), where the point is that only reading code does not give you the ability to successfully execute the instructions. But this is also a lack of experience that can be remedied completely within the textual world, and hence does not strengthen the point of Bisk et al. (2020). Factoring in that two entities that share the same set of modalities do not necessarily share any experiences, we find the assumption that text is an insufficient medium for gathering shared experiences to be lacking rigorous backing.

As previously stated, we are in favor of integrating multiple modalities into our language AI systems. This seems to be by far the most viable approach for incorporating and accumulating shared experiences. The distinction is that we are not convinced by the proposed theoretical requirements on additional modalities. We are also greatly in favor of the notion that the communication with the intelligent octopus (or any other entity) only has meaning because meaning is being inferred. Indeed we wish to push this notion even further than Bender and Koller (2020), as we believe that this concept is key to this discussion. This is elaborated upon in Section 4.2.

### 3.2 Communicative Intent and the Cartesian Theater

Even if we disagree that the octopus test disproves that language models actually understand language, we *do* think it points to one important aspect of language use: namely that of *agency*. That is, if O is only a language model, we would not expect it to spontaneously reply to some statement from A or B, since it has no incentives to do so. A language model only produces an output when prompted; it has no will or intent of its own. Human language users, by contrast, have plans, ambitions and intents, which drive their linguistic actions. We humans play language games in order to achieve some goal, e.g., to make someone open a door, or to insult someone by giving them an instruction they cannot complete. Language models (and other current NLU techniques) *execute* language games when prompted to do so, but the intent is typically supplied by the human operator.

Of course, there is one specific NLP application that is specifically concerned with intents: dialogue systems (or chatbots, to use the more popular vernacular). Consider a simple chatbot that operates after a given plan, for example to call a restaurant and book a table for dinner. Such a chatbot would not only be able to act according to its own intents, but would presumably also be able to recognize its interlocutor’s intents (by simply classifying user responses according to a set of given intent categories). If we were to take the position that pragmatics is the necessary requirement for understanding, as Bender and Koller (2020) seems to do, it would lead to the slightly odd consequence that a simple (perhaps even completely scripted) dialogue system would count as having a fuller understanding of language than a language model that is capable of near-human performance on reading comprehension tasks. Such a comparison is of course nonsensical, since these systems are designed to perform different types of tasks with different linguistic requirements and thus cannot be compared on a single scale of understanding (there is no such thing).

This reductio ad absurdum example demonstrates the perils of strict definitions, such as the *M* ⊆ *E* × *I* formula for meaning suggested by Bender and Koller (2020).^2^ The main problem with translating intentions in mathematical terms is that the concept is at best very vague. In operationalizations of intent recognition (e.g., in chatbots), we operate with a limited and predefined taxonomy of intents that are relevant to a specific use case, but in open language use it is less clear how to assign intents to expressions. At what granular level do intents reside—is it at the word level, sentence level, or speaker turn (and how does that translate into text, where a turn sometimes is an entire novel)? And is the subject always in a privileged position to identify her intents? This is questioned in particular by postmodern critical theory (Pluckrose and Lindsay, 2020), and there are plenty of examples in the public debate where the speaker’s interpretation of her utterance differs from that of commentators (“I did not mean it like that” is not an uncommon expression). This lends a certain hermeneutic flavor to the concept of intent, which makes it slightly inconvenient to use in mathematical formulas.

We believe that pragmatics is no less, and arguably even more, a product of conventionalization processes in language use than other types of understanding. This may be an uncontroversial statement, but it points to a question that certainly is not, namely whether pragmatic understanding can be acquired by only observing the linguistic signal. Bender and Koller (2020) clearly think not, and they argue that grasping intents requires extralinguistic knowledge. For a simple case such as “door!”, this may entail being able to recognize the object referred to, and possibly also knowledge about how it operates. For more abstract concepts, Bender and Koller’s claim the existence of “abstract” or “hypothetical world(s) in the speaker’s mind.” There is an apparent risk that the invocation of minds at this point collapses into Cartesian materialism (Dennett, 1991), which constructs the mind as a kind of control room (also referred to as a “Cartesian theater”) where the subject—the *self* or *homunculus*—observes, interprets, and controls the outside world. We are not sure what Bender and Koller (2020) position would be with respect to such a view, but it is easy to see how it would posit the intents with the homunculus, which would then use the linguistic generator to express its intents in the form of language—which is, in our understanding, more or less exactly what Bender and Koller (2020) propose.

### 3.3 How the Current Critique Rekindles Distributionalism

The distinction between *form* (i.e., the linguistic signal) and *meaning* (i.e., the intent) is central to Bender and Koller (2020), and they claim that a model (or more general, a subject) “trained purely on form will not learn meaning”. But if meanings can have an effect on the form (which we assume everyone agrees on), then a model should at least be able to observe, and learn, these effects. The point here is that there needs to be an accessible “linguistic correlate” to whatever meaning process we wish to stipulate, since otherwise communication would not be possible. Thus, in the sense that intentions (meanings) have effects on the linguistic signal (form), it will be possible to learn these effects by simply observing the signal. It is precisely this consideration that underlies the distributional approach to semantics. Harris (1954) provides the most articulate formulation of this argument: “As Leonard Bloomfield pointed out, it frequently happens that when we do not rest with the explanation that something is due to meaning, we discover that it has a formal regularity or “explanation.” It may still be “due to meaning” in one sense, but it accords with a distributional regularity.”

Somewhat ironically, Bender and Koller’s objections to distributional approaches in the form of language models—that meaning is something unobtainable from simply observing the linguistic signal—thus effectively brings us back to the original motivation for using distributional approaches in computational linguistics in the first place: if meanings are unobtainable from the linguistic signal, then all we can do from the linguistic perspective is to describe the linguistic regularities that are manifestations of the external meanings.

This motivation for distributional approaches seems to completely elude Bender and Koller, who devote one section of their paper to distributional semantics, but fail to relate to this line of reasoning. Instead, they quote the “Wittgensteinian” slogan “meaning is use”^3^ and claim that the “use” in this slogan does not refer to textual distributions. This is irrelevant for the distributional hypothesis, but it is certainly true if we are to adopt a purely Wittgensteinian perspective on meaning and use. For Wittgenstein, language use and meaning relates intimately to the notion of “form of life,” which is the common (historical, cultural, etc.) context shared by human language users, and is the ultimate enabler of communication and meaning. In a strict reading of this idea, we simply cannot understand the meaning of an expression unless we share the form of life of the speaker. As Wittgenstein famously put it: “if a lion could talk, we wouldn’t be able to understand it” (Wittgenstein, 1953). This line of reasoning then leads to a position where understanding (of human meaning) is only possible by other humans who share the specific contextual conditions of the speaker. This in turn means that the prospect of NLU is futile and ultimately doomed to fail unless we can build an entire human in silico. Compared to such a defeatist position, distributionalism seems like a wonder of potential.

## 4 At the End of the Ascent

In the last section, we argued that the currently dominating approach in NLU—distributionally-based methods—originate in a reaction against precisely the kind of dualism professed by Bender and Koller (2020). But even if the motivation for the current main path in NLU thereby should be clear, the question still remains whether this current path is feasible in the long run, or whether it will eventually lead to a dead-end. This section discusses what the dead-end might look like, and what that means for the hill-climbing question.

### 4.1 The Chinese Room and Philosophical Zombies

Bender and Koller’s main concern seems to be that our current research direction in NLU will lead to something like a Chinese Room. The Chinese Room argument is one of the classical philosophical thought experiments, in which Searle (1980) invites us to imagine a container (such as a room) populated with a person who does not speak Chinese, but who has access to a set of (extensive) instructions for manipulating Chinese symbols, such that when given an input sequence of Chinese symbols, the person can consult the instructions and produce an output that for a Chinese speaker outside the room seems like a coherent response. In short, the Chinese Room is much like our current language models. The question is whether any real understanding takes place in the symbol manipulating process?

We will not attempt to contribute any novel arguments to the vast literature that exists on the Chinese Room argument, but we will point to the counter-argument commonly known as the “Systems Reply” (Searle, 1980). This response notes that for the observer of the room (whether it is an actual room, a computer, or a human that has internalized all the instructions) it will seem *as if* there is understanding—or at least language proficiency—going on in the room. Similarly, for the user of a future NLU system (and perhaps even for certain current users of large-scale language models), it may seem as if the system understands language, even if there is “only” a language model on the inside. We can of course always question whether there is any “real” understanding going on, but if the absence or presence of this “real” understanding has no effect on the behavior of the system, it will be a mere epiphenomenon that need not concern us. This is a variant of the Systems Reply, and is essentially the same argument as the Philosophical Zombies [or Zimboes, as Dennett calls them (Dennett, 1998)] that behave exactly like human beings, except that they have no consciousness. Such a being would not be able to *really* “want,” “believe,” and “mean” things, but we would probably still be better off using these terms to explain their behavior.

### 4.2 Understanding and the Intentional Stance

This is what Dennett refers to as “Intentional Stance” (Dennett, 1987): we ascribe intentionality to a system (or more generally, entities) in order to explain and predict its behavior. For very basic entities, such as a piece of wood, it is normally sufficient to ascribe physical properties to it in order to explain its behavior (it has a certain size and weight, and will splinter if hit by a sharp object). For slightly more complex entities, such as a chainsaw, we also ascribe functions that explain its expected behavior (if we pull the starter cord, the chain will start revolving along the blade, and if we put it against a piece of wood, it will saw through the wood). For even more complex entities, such as animals and human beings, it is normally not enough with physical properties and functional features to explain and predict their behavior. We also need to invoke intentionality—i.e., mental capacities—in order to fully describe them. Note that we occasionally do this also with inanimate objects, in particular when their behavior starts to deviate from the expected functions: “the chainsaw doesn’t want to start!” We are in such cases not suggesting that the chainsaw suddenly has become conscious in the same way as humans are conscious; it is simply more convenient to adopt an intentional stance in the absence of simpler functional explanations. It would probably be possible to provide purely functional, perhaps even mechanistic explanations at some very basic neurophysiological level for every action that an animal or human makes, but it would be quite cumbersome. The intentional stance is by far the more convenient perspective.

Dennett’s point is that consciousness is not an extra ingredient in addition to the complexity of a system: consciousness *is* the complexity of the system. Our point is that understanding is also not an extra ingredient of a symbol manipulation system: “understanding” is a term we use to describe the complexity of such a system. When the behavior of an NLU system becomes sufficiently complex, it will be easier to explain its behavior using intentional terms such as “understanding,” than to use a purely functional explanation. We posit that we are not far from a future where we habitually will say that machines and computer systems understand and misunderstand us, and that they have intents, wishes, and even feelings. This does not necessarily mean that they have *the same type* of understanding, intents, opinions, and feelings as humans do, but that their behavior will be best explained by using such terms. The same situation applies to animals (at least for certain people), and maybe even to plants (with the same caveat). We argue that the question whether there really is understanding going on, i.e., whether there is also some mapping process executed in addition to the language use or behavior, is redundant in most situations. We can probably look forward to interesting and challenging ethical and philosophical discussions about such matters in the future, but for most practical purposes, it will be of neither interest nor consequence to question whether an NLU system is a mere symbol manipulator or a “true” understander.

To be fair, Bender and Koller (2020) foresee a counterargument along these lines, which is essentially a mix between the arguments listed by the authors as “But aren’t neural representations meaning too?” and “But BERT improves performance on meaning-related tasks, so it must have learned something about meaning.” Interestingly, Bender and Koller (2020) concede that both of these possible counterarguments are at least partially valid (albeit to a very modest degree), but in both cases they eventually dismiss the counterarguments by invoking the concept of “actual meaning.” As should be obvious by now, we believe there is no such thing, and that the invocation of this concept is an instance of the singleton fallacy. We do share, however, the sneaking suspicion that we are not yet fully done in the development of NLU solutions, and that the current generation of “Muppetware” (BERT, ERNIE, ELMo, Big Bird, etc.) is only the beginning of much more interesting things to come.

### 4.3 Montology

It is important to understand which hill we are currently climbing, and why. As we have argued in this paper, the current hill, on which language models and most current NLU approaches live, is based on distributional sediment, which has amassed as a reaction to a dualistic view that posits understanding and meaning in a mental realm outside of language. The purpose of this hill is not to replicate a human in silico, but to devise computational systems that can manipulate linguistic symbols in a manner similar to humans. This is not the only hill in the NLU landscape—there are hills based on logical formalisms, and hills based on knowledge engineering—but based on the empirical evidence we currently have, the distributional hill has so far proven to be the incomparably most accessible ascent.

It should thus come as no surprise that our answer to the question whether we are climbing the right hill is a resounding *yes*. We do, however, agree that we should not only climb the most accessible hills, and that we as a field need to encourage and make space for alternative and complementary approaches that may not have come as far yet. The most likely architecture for future NLU solutions will be a combination of different techniques, originating from different hills of the NLU landscape.

## 5 Conclusion

This paper has argued that much of the current debate on language models rests on what we have referred to as the singleton fallacy: the assumption that language, meaning, and understanding are single and uniform phenomena that are unobtainable by (current) language models. By contrast, we have argued that there are many different types of language use, meaning and understanding, and that (current) language models are built with the explicit purpose of acquiring and representing one type of structural understanding of language. We have argued that such structural understanding may cover several different modalities, and as such can handle several different types of meaning. Importantly, we see no theoretical reason why such structural knowledge would be insufficient to count as understanding. On the contrary, we believe that as our language models and NLU systems become simultaneously more proficient and more complex, users will have no choice but to adopt an intentional stance to these systems, upon which the question whether there is any “true” understanding in these systems becomes redundant.

We are well aware that the current debate is of mainly philosophical interest, and that the practical relevance of this discussion is small to non-existent. A concrete suggestion to move the discussion forward is to think of ways to verify or falsify the opposing positions. We distinctively feel that the burden of proof lies with the opposition in this case; are there things we cannot do with language unless we have “real” (as opposed to structural) understanding? Which criteria should we use in order to certify NLU solutions as being “understanders” rather than mere symbol manipulators? Being able to solve our current General Language Understanding Evaluation benchmarks (i.e., GLUE and SuperGLUE) obviously does not seem to be enough for the critics.

From the ever-growing literature on “BERTology” (Rogers et al., 2020) we know that there are tasks and linguistic phenomena that current language models handle badly, if at all, and we also know that they sometimes “cheat” when solving certain types of tasks (Niven and Kao, 2019). These are extremely valuable results, which will further the development of language models and other types of NLU solutions. However, these failures do not mean that language models are theoretically incapable of handling these tasks; it only means that our *current* models (i.e., current training objectives, architectures, parameter settings, etc.) are incapable of recognizing certain phenomena. Which, we might add, is to be expected, given the comparably simple training objectives we currently use.

## Footnotes

1 In response to Bender and Koller (2020) prompting this question to GPT-2, we prompted this question to our human colleges. The responses ranged from *“Throw the sticks at the bear”*, *“Do bears really live on islands?”*, *“End your suffering, bear is gonna win anyway”* to *“Drinking on the job again are we?”*. As we find it unlikely that any of these answers adequately solves the imminent bear problem, we hence conclude that none of our colleagues possess natural language understanding. We do however find it likely that a sophisticated language model could produce similar answers.

2 *M* is meaning, *E* is expression, and *I* is intent.

3 Wittgenstein’s exact formulation is “For a large class of cases—though not for all—in which we employ the word “meaning” it can be defined thus: the meaning of a word is its use in the language” (Wittgenstein, 1953).

## References

[B1] Bender E. M. Koller A. (2020). “Climbing Towards NLU: On Meaning, Form, and Understanding in the Age of Data,” in Proceedings of the 58th Annual Meeting of the Association for Computational Linguistics (Online: Association for Computational Linguistics), 5185–5198. 10.18653/v1/2020.acl-main.463

[B3] Bisk Y. Holtzman A. Thomason J. Andreas J. Bengio Y. Chai J. (2020). “Experience Grounds Language,” in Proceedings of the 2020 Conference on Empirical Methods in Natural Language Processing (EMNLP) (Online: Association for Computational Linguistics), 8718–8735. 10.18653/v1/2020.emnlp-main.703

[B4] Brown T. Mann B. Ryder N. Subbiah M. Kaplan J. D. Dhariwal P. (2020). “Language Models are Few-Shot Learners,” in Advances in Neural Information Processing Systems. Editors Larochelle H. Ranzato M. Hadsell R. Balcan M. F. Lin H. (Curran Associates, Inc.), Vol. 33, 1877–1901.

[B5] Collobert R. Weston J. (2008). “A Unified Architecture for Natural Language Processing,” in Proceedings of the 25th International Conference on Machine Learning (New York, NY, USA: Association for Computing Machinery), 160–167. 10.1145/1390156.1390177

[B6] Dennett D. C. (1987). The Intentional Stance. Cambridge, MA: The MIT Press.

[B7] Dennett D. (1998). “The Unimagined Preposterousness of Zombies,” in Brainchildren: Essays on Designing Minds (The MIT Press). 10.7551/mitpress/1663.003.0015

[B8] Dennett D. (1991). Consciousness Explained (Little, Brown).

[B9] Devlin J. Chang M.-W. Lee K. Toutanova K. (2019). “BERT: Pre-Training of Deep Bidirectional Transformers for Language Understanding,” in Proceedings of the 2019 Conference of the North American Chapter of the Association for Computational Linguistics: Human Language Technologies (Minneapolis, Minnesota: Association for Computational Linguistics), 4171–4186. 10.18653/v1/N19-1423

[B10] Ettinger A. (2020). What BERT Is Not: Lessons From a New Suite of Psycholinguistic Diagnostics for Language Models. Trans. Assoc. Comput. Linguistics. 8, 34–48. 10.1162/tacl_a_00298

[B11] Gastaldi J. L. (2020). Why Can Computers Understand Natural Language? The Structuralist Image of Language Behind Word Embeddings. Philos. Technol. 34, 149–214. 10.1007/s13347-020-00393-9

[B12] Harris Z. S. (1954). Distributional Structure. WORD 10, 146–162. 10.1080/00437956.1954.11659520

[B13] Herdade S. Kappeler A. Boakye K. Soares J. (2019). “Image Captioning: Transforming Objects into Words,” in Advances in Neural Information Processing Systems. Editors Wallach H. Larochelle H. Beygelzimer A. d’ Alché-Buc F. Fox E. Garnett R. (Curran Associates, Inc.), Vol. 32.

[B14] Jawahar G. Sagot B. Seddah D. (2019). “What Does BERT Learn About the Structure of Language?,” in Proceedings of the 57th Annual Meeting of the Association for Computational Linguistics (Florence, Italy: Association for Computational Linguistics), 3651–3657.

[B15] Li G. Zhu L. Liu P. Yang Y. (2019). “Entangled Transformer for Image Captioning,” in Proceedings of the IEEE/CVF International Conference on Computer Vision (ICCV). 10.1109/iccv.2019.00902

[B16] Liu N. F. Gardner M. Belinkov Y. Peters M. E. Smith N. A. (2019). “Linguistic Knowledge and Transferability of Contextual Representations,” in Proceedings of the 2019 Conference of the North American Chapter of the Association for Computational Linguistics: Human Language Technologies (Minneapolis, Minnesota: Association for Computational Linguistics), 1073–1094. 10.18653/v1/n19-1112

[B17] Marr B. (2020). What is GPT-3 and Why is it Revolutionizing Artificial Intelligence? Forbes.

[B18] McCoy T. Pavlick E. Linzen T. (2019). “Right for the Wrong Reasons: Diagnosing Syntactic Heuristics in Natural Language Inference,” in Proceedings of the 57th Annual Meeting of the Association for Computational Linguistics (Florence, Italy: Association for Computational Linguistics), 3428–3448. 10.18653/v1/p19-1334

[B19] Nie Y. Williams A. Dinan E. Bansal M. Weston J. Kiela D. (2020). “Adversarial NLI: A New Benchmark for Natural Language Understanding,” in Proceedings of the 58th Annual Meeting of the Association for Computational Linguistics (Online: Association for Computational Linguistics), 4885–4901. 10.18653/v1/2020.acl-main.441

[B20] Niven T. Kao H.-Y. (2019). “Probing Neural Network Comprehension of Natural Language Arguments,” in Proceedings of the 57th Annual Meeting of the Association for Computational Linguistics (Florence, Italy: Association for Computational Linguistics), 4658–4664. 10.18653/v1/P19-1459

[B21] Peters M. E. Neumann M. Iyyer M. Gardner M. Clark C. Lee K. (2018). “Deep Contextualized Word Representations,” in Proceedings of the 2018 Conference of the North {A}merican Chapter of the Association for Computational Linguistics: Human Language Technologies, Volume 1 (Long Papers), New Orleans, LA (Association for Computational Linguistics), 2227–2237. 10.18653/v1/N18-1202

[B22] Pluckrose H. Lindsay J. (2020). Cynical Theories: How Activist Scholarship Made Everything about Race, Gender, and Identity–And Why This Harms Everybody. Pitchstone Publishing.

[B23] Radford A. Kim J. W. Hallacy C. Ramesh A. Goh G. Agarwal S. (2021). “Learning Transferable Visual Models From Natural Language Supervision,” in Proceedings of the 38th International Conference on Machine Learning (ICML), Virtual Event, July 18–24, 2021 (PMLR), Vol. 139, 8748–8763. Available at: http://proceedings.mlr.press/v139/radford21a.html.

[B24] Ramesh A. Pavlov M. Goh G. Gray S. Chen M. Child R. (2021). DALL·E: Creating Images from Text. Available at: https://openai.com/blog/dall-e/.

[B25] Ribeiro M. T. Wu T. Guestrin C. Singh S. (2020). “Beyond Accuracy: Behavioral Testing of NLP Models With CheckList,” in Proceedings of the 58th Annual Meeting of the Association for Computational Linguistics (Online: Association for Computational Linguistics), 49024912. 10.18653/v1/2020.acl-main.442

[B26] Rogers A. Kovaleva O. Rumshisky A. (2020). A Primer in BERTology: What We Know about How BERT Works. Trans. Assoc. Comput. Linguistics. 8, 842–866. 10.1162/tacl_a_00349

[B27] Sahlgren M. (2008). The Distributional Hypothesis. Ital. J. Linguistics. 20, 33–54.

[B28] Searle J. R. (1980). Minds, Brains, and Programs. Behav. Brain Sci. 3, 417–424. 10.1017/s0140525x00005756

[B29] Su W. Zhu X. Cao Y. Li B. Lu L. Wei F. (2020). “Vl-Bert: Pre-Training of Generic Visual-Linguistic Representations,” in International Conference on Learning Representations.

[B30] Tenney I. Xia P. Chen B. Wang A. Poliak A. McCoy R. T. (2019). “What Do You Learn From Context? Probing for Sentence Structure in Contextualized Word Representations,” in International Conference on Learning Representations.

[B31] Thornhill J. (2020). Is {AI} Finally Closing in on Human Intelligence? Financial Times. Available at: https://www.ft.com/content/512cef1d-233b-4dd8-96a4-0af07bb9ff60.

[B32] Wang A. Pruksachatkun Y. Nangia N. Singh A. Michael J. Hill F. (2019). “Superglue: A Stickier Benchmark for General-Purpose Language Understanding Systems,” in Advances in Neural Information Processing Systems. Editors Wallach H. Larochelle H. Beygelzimer A. d’Alché Buc F. Fox E. Garnett R. (Curran Associates, Inc), 32, 3266–3280.

[B33] Wang A. Singh A. Michael J. Hill F. Levy O. Bowman S. (2018). “GLUE: A Multi-Task Benchmark and Analysis Platform for Natural Language Understanding,” in Proceedings of the 2018 EMNLP Workshop BlackboxNLP: Analyzing and Interpreting Neural Networks for NLP (Brussels, Belgium: Association for Computational Linguistics), 353–355. 10.18653/v1/w18-5446

[B35] Wittgenstein L. (1953). Philosophical Investigations. Oxford: Basil Blackwell.

[B34] Yin W. Hay J. Roth D. (2019). “Benchmarking Zero-shot Text Classification: Datasets, Evaluation and Entailment Approach,” in Proceedings of the 2019 Conference on Empirical Methods in Natural Language Processing and the 9th International Joint Conference on Natural Language Processing (EMNLP-IJCNLP), Hong Kong, China (Association for Computational Linguistics), 3914–3923. 10.18653/v1/D19-1404

